# Immune response during acute Chandipura viral infection in experimentally infected susceptible mice

**DOI:** 10.1186/1743-422X-5-121

**Published:** 2008-10-20

**Authors:** Anukumar Balakrishnan, Akhilash Chandra Mishra

**Affiliations:** 1 Chandipura virus group, National Institute of Virology, 20-A, Dr. Ambedkar Road, Post Box-11, Pune-411001, Maharashtra, India; 2 Director, National Institute of Virology, 20-A, Dr. Ambedkar Road, Post Box-11, Pune-411001, Maharashtra, India

## Abstract

**Background:**

Age dependent susceptibility was observed in Chandipura virus (CHPV) infected mice through intravenous and intraperitoneal route. Adult mice were susceptible only through intracerebral route of infection. Immature neuron and some other biological variables including immature immune system are considered to be important factor for age related susceptibility in some diseases. As Chandipura virus infects both young and adult mice brain through intracerebral route the role of immune system during peripheral infection in young susceptible mice needs to be studied.

**Results:**

Through intravenous route of infection the virus produces vireamia and cross the blood brain barrier (BBB) to replicate in the central nervous system. Circulating virus is effectively cleared by virus specific IgM antibody but replication in CNS continues. The infected mice secreted significant amount of proinflammatory cytokines like TNFα and MCP-1 and high amount of IFNγ, IL-1 and IL-6 at 24 h post infection. Reduction in significant amount of CD4, CD8 and CD19 positive cells at 72 h post infection (p < 0.000) was observed in infected mice. Suppression of T cell proliferation of splenocytes to Con A (p < 0.000), LPS and specific antigen was also observed. Presence of preformed virus specific antibody in the form of passive immunization completely protected the mice but immunization on the day or after the virus infection could not completely protect the mice.

**Conclusion:**

Proinflammatory cytokines at 24 h post infection and reduction of CD4, CD8 and CD19 positive immune cells might make the mice immune compromised during infection. These cytokines might also increase the permeability of BBB to allow the virus to enter into CNS. Virus replication in CNS is responsible for neurological symptom and mortality. Once virus gets established in CNS it is difficult to protect the mice by passive immunization.

## Background

The association of Chandipura virus with acute encephalitis outbreak in Andra Pradesh, Maharashtra and Gujarat clearly attributed the disease potential of this virus [1,24]. Children below 15 years old were vulnerable but adults were refractory to the infection. Symptoms of high grade fever, vomiting, altered sensorium, generalized convulsions, decerebrate posture and grade IV coma was noticed in hospitalised children. Children died within 48 h of hospitalization. Age dependent susceptibility of Chandipura virus in murine model was reported by several authors [2-4]. Although age dependent susceptibility noticed in several neurotropic viruses, including rhabdoviurses, reoviruses, bunyaviruses, alphaviruses and flaviviruses [5-10], the mechanisms involving age dependent resistance to fatal viral encephalitis have been largely inconclusive. Studies on Semiliki forest virus, Sindbis virus, Japanese encephalitis virus [11] and reovirus [12] concluded that the neuronal maturation is a critical factor for resistance to viral infection. Other biological variables like maturation of the reticuloendothelial system [14], development of anatomic barriers [15], changes in receptor availability [16], potentiation of interferon (IFN) production [17], acceleration of immune responses [18,19], and decreases in systemic stress responses [20] are other factors. Labrada *et al*, 2002 described that the novel interferon inducible protective gene (ISG12) delay the Sindbis virus induced death in neonatal mouse [21]. In a broad sense the mechanism(s) might be either due to the host immune response against the viral infection or the virus tropism in central nervous system or combination of both.

Chandipura virus is lethal to young mice by peripheral as well as central route of infection but adult mice are susceptible only through central route of infection [3]. Thus immature neuron is not a critical factor for Chandipura virus pathogenesis. The role of immune response during infection is not understood. Present study was undertaken to understand role of innate, humoral and cell mediated immune response in experimentally infected susceptible mice through intravenous route of infection.

## Results

### Pathogenesis in mice

**Figure 1:**
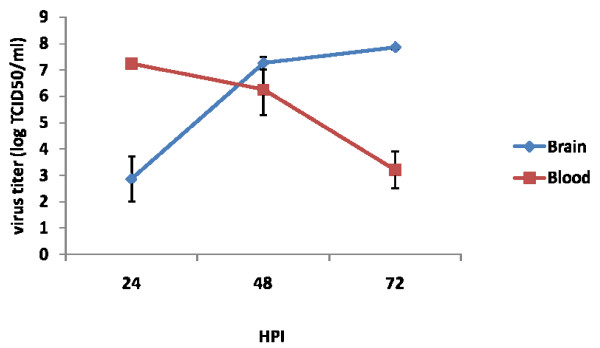
**Virus titer in blood and brain from mice at different hours post infection (HPI)**. The serum and brain supernatant from infected as well as control mice was titrated in Vero E6 cells. The end point was determined by the reciprocal of highest dilution which showing CPE. The tissue culture infective dose 50 (TCID_50_) per ml (TCID50/ml) was calculated by Reed and Munch formula. The values are Mean ± SE of two experiments. Each experiment tissues were collected from three different mice from each group and processed separately.

In blood at 24 h post infection (PI) the titer was log 7.25 ± 0.045 then it was reduced to log 3.19 ± 0.7 at 72 h PI. However in brain maximum titer was noticed at 72 h PI (log7.85 ± 0.07) and then most of the mice died. Initially at 24 h PI the titer in the brain was log 2.85 ± 0.85. At 48 h PI the titer in blood and brain was almost similar with titer of 6.25 ± 0.97 and 7.25 ± 0.25 respectively (Fig. 1).

**Figure 2:**
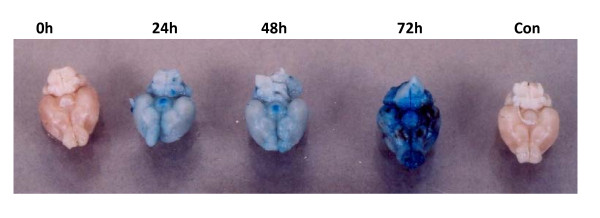
**Blood Brain Barrier damage of mice infected with virus by Evan's Blue dye exclusion test**. Inclusion of blue colour dye in the brain indicated the increase permeability or damage of BBB. The picture showed here was selected brain from different hours post infection of three experiments.

Chandipura virus infected susceptible mice the leaking of Evan's blue dye in brain started from 24 h PI onwards. At 72 h PI the intensity was very high and indicated more damage or permeability of BBB (Fig. 2).

### Humoral immune response

**Figure 3:**
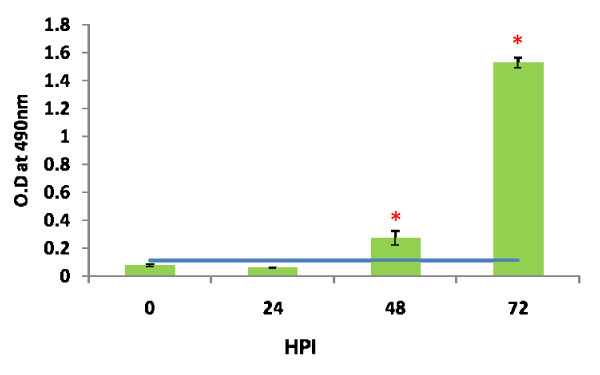
**A_490 _of Chandipura specific IgM in sera at different hours post infection (HPI)**. The level of IgM in the sera was determined by mouse IgM capture ELISA. The values are optical density (O.D) at 490 nm wave length. The serum from three mice of infected as well as uninfected mice was processed separately in each post infective hours. The values are Mean ± SE of three experiments. Cut off value derived from mean O.D of age matched uninfected control mice plus 3 SD. *p < 0.05

Based on the cut off value (0.112) the IgM sero conversion was noticed at 48 h PI with O.D. of 0.270 ± 0.05. At 72 h PI the O.D. (1.526 ± 0.038) was significantly higher than 24 (0.056 ± 0.001) and 48 h PI (*p *< 0.05) (Fig. 3). All the samples were negative for Chandipura virus specific IgG antibody.

**Figure 4:**
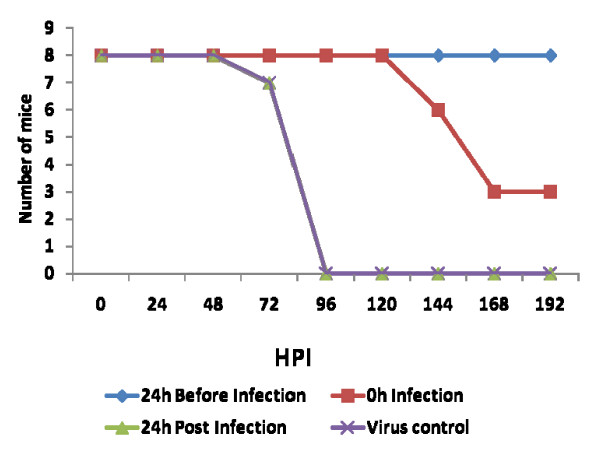
**Survival pattern of mice passive immunized with chandipura virus specific antibody**. Mice in group were immunized with rabbit anti Chandipura antibody at different time point viz before infection, along with infection and after infection. The immunization was continued upto 72 h PI. Virus control without immunization and uninfected control was also kept along with immunized group. The survivabilty was observed for 8 days post infection. The graph is representative of three independent experiments.

The mice immunized with anti Chandipura antibody 24 h before virus infection survived with no gross symptom. Partial protection around 20–30% was observed in mice simultaneously infected as well as immunized. Those mice that escaped death showed neurological symptom like hind limb paralysis. The passive immunization could not protect the mice immunized 24 h PI (Fig. 4).

### Cell mediated immune response

**Figure 5:**
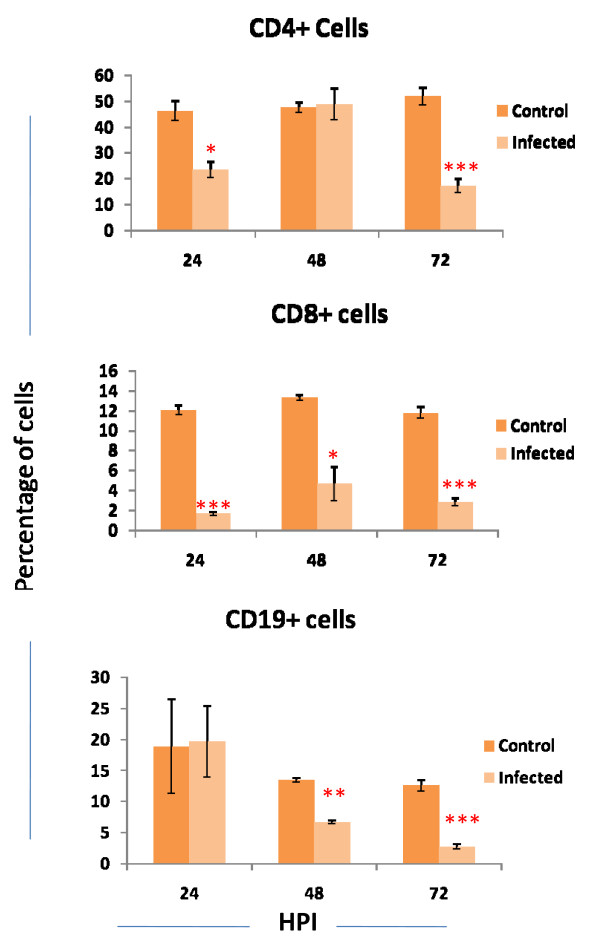
**Percentage of CD4, CD8 and CD19 positive cells in blood of infected as well as control mice at different hours post infection (HPI)**. Total 10000 cells were acquired and lymphocyte population was gated in FSC *vs *SSC dot plot. From the gated population the percentage of CD4, CD8 and CD19 positive cell were calculated by cell quest software. The values are Mean ± SE of three independent experiments. Each experiments two mice from infected and control mice were processed separately. *p < 0.05, **p < 0.01, ***p < 0.000.

The percentage of CD4+ cells in infected mice at 24 h PI was significantly lower than uninfected control mice (23.41 ± 3.16 vs 46.28 ± 4.01). Similarly severe reduction was noticed (17.2 ± 2.74 vs 51.9 ± 3.4) at 72 h PI. In CD8+ cell significant reduction was noticed in variably in all PI days (*p *< 0.05). The values ranging from 1.62 ± 0.21 at 24 h PI to 2.785 ± 1.735 at 72 h PI compared to the control which was 12.06 ± 0.48 at 24 h PI to 11.77 ± 0.6 at 72 h PI. In CD19+ cells significant reduction was noticed at 48 h (6.73 ± 0.34 vs 13.45 ± 0.34) and 72 h PI (2.75 ± 0.415 vs 12.61 ± 0.915) (Fig. 5).

**Figure 6:**
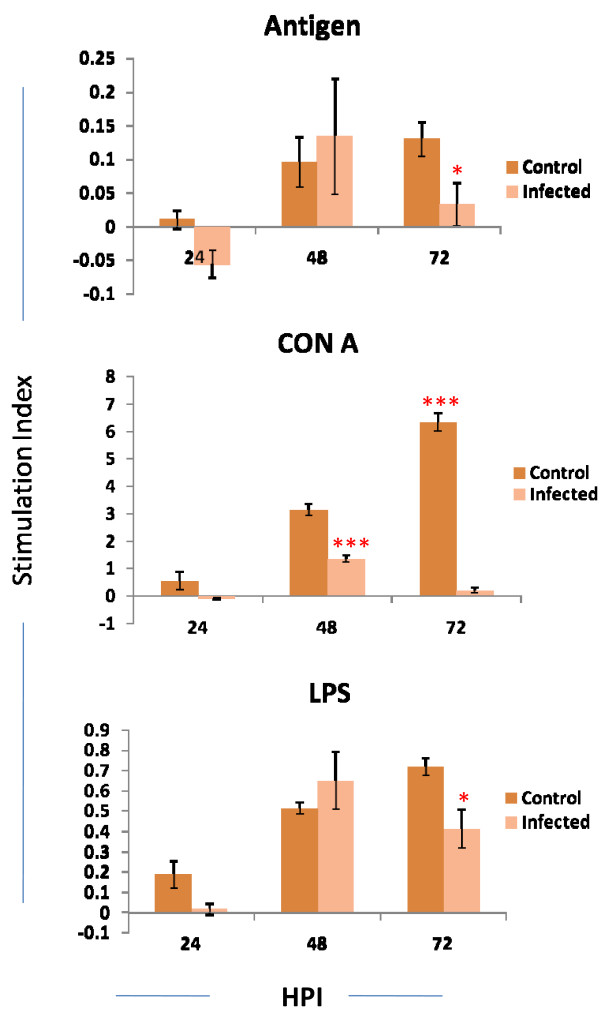
**Stimulation index (SI) of splenocytes from infected and control mice stimulated with Concanavalin A, LPS and BPL inactivated viral antigen**. The end point was determined by colorimetric MTT dye reduction test. The stimulation index was calculated by O.D of stimulated-O.D of unstimulated/O.D. of unstimulated. The stimulation index with difference of two from control mice was kept as a cut off value. Those mice showing cut off value above than control mice was considered proliferation and the value below two was considered as suppression. The values are Mean ± SE of two independent experiments. Each experiment two individual spleen from infected and control mice was processed separately. *p < 0.05, *** *p *< 0.000.

Significant antigen specific suppression of T cell proliferation was noticed in the splenocytes from infected mice at 72 h PI compared to the control (Stimulation Index (SI) 0.057 ± 0.017 vs 0.129 ± 0.026). Similarly Con A mediated suppression of T cell proliferation was noticed at 48 h and 72 h PI with SI of 1.363 ± 0.14 vs 3.147 ± 0.216 and 0.22 ± 0.098 vs 6.336 ± 0.33 respectively. The suppression was highly significant (*p *< 0.000). Significant LPS mediated suppression was noticed at 24 h PI (SI 0.017 ± 0.028 vs 0.251 ± 0.026) (Fig. 6).

### Innate immune response

**Figure 7:**
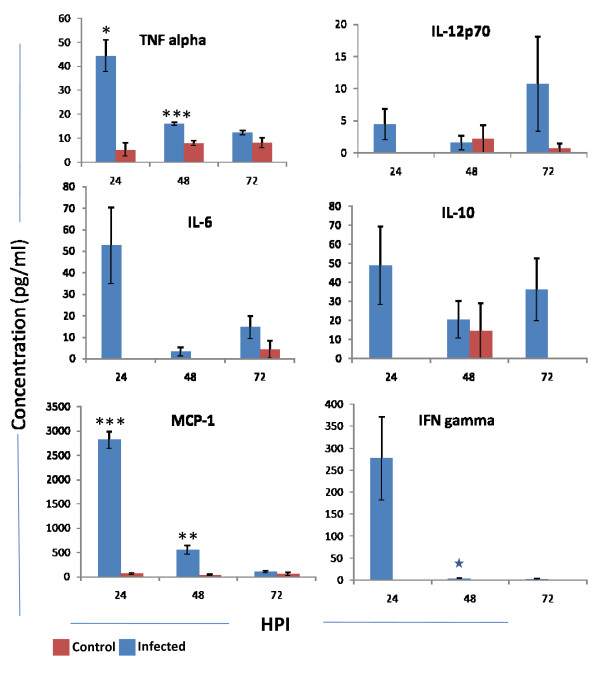
**Level of proinflammatory cytokines (TNFα, IFNγ, MCP-1, IL-6, IL-10 and IL-12p70) in plasma of mice at different hours post infection (HPI)**. At various time point three mice from both infected as well as control mice were bled intra orbitaly in EDTA. The plasma was separated by centrifugation and 50 μl was used for quantitation of cytokines. The cytokines were quantitated by using mouse inflammatory beads assay. The sensitivity of this assay is 0–5000 pg/ml. The values are Mean ± SE of three experiments. Each experiment plasma from three mice was processed separately at different post infective hours. *p < 0.05, **p < 0.01, *** *p *< 0.000.

At 24 h PI significant (*p *< 0.05) quantity of TNFα was noticed (44.42 ± 6.64) and it was reduced to 12.4 ± 0.93 at 72 h PI. Similar pattern was also noticed in MCP-1 with 2815.9 ± 177.48 pg/ml at 24 h PI and 104.46 ± 19.71 pg/ml at 72 h PI. At 24 h PI quantity of cytokines like IFNγ, IL-10 and IL-6 (277.25 ± 95.32, 48.72 ± 20.50 and 52.55 ± 17.90) were very high compared to the other post infective hours. In contrary IL-12p70 was high at 72 h PI (10.69 ± 7.4) (Fig. 7).

## Discussion

In young mice through intravenous injection this virus crosses the BBB and infects the CNS. In this study we observed high virus titer at 72 h PI in brain and death occurred between 72 and 96 h PI. Jortner *et al *(1972) reported rising viral titers in blood, skeletal muscle and viscera, beginning 3 to 6 h PI of intra peritonealy (I/P) inoculated 9-day old mice, significant quantities of virus in the brain at 24 h PI, neurologic dysfunctions at 48 h PI and death between 72 and 96 h PI.

In blood rapid replication of virus with maximum titer at 24 h PI was noticed. However the titer declined at 72 h PI. Simultaneously the IgM also was noticed in blood. The production of virus specific IgM at 48 h PI correlates with clearance of virus from the circulation. The rising antibody level in blood did not correlate with clearance of virus from the CNS. The same phenomenon was also observed in Semiliki forest virus infection in mice [26]. The authors described that IgM antibody control vireamia but are unable to eliminate the CNS infection. In infected mice the preformed antibody in the circulation by passive immunization protected the mice by neutralizing the virus. The passive immunization at 24 h PI did not help to protect the mice. Because virus in brain at 24 h PI indicated that the virus might crosses the BBB within 24 h PI. The antibody might not penetrate enough to deep into the CNS to clear the virus from neuron. When the mice were infected with virus simultaneously immunized with antibody the protection was partial. This might be due to the intra peritonealy delivered antibody may take time to reach the circulation to clear the virus. It was concluded that once the virus enters the CNS, antibody may not effective to control the pathogenesis. After several experiments still we couldn't get consistent results of BBB permeability by Evan's blue dye exclusion test. This might be due to transient changes in BBB during infection. These permeability changes in BBB might be contributed by several factors during infection. Proinflammatory cytokines are one of the main factor indicated in several studies especially TNFα [27,28]. In this study we observed that at 24 h PI presence of high quantity of proinflammatory cytokines like TNFα, IFNγ, IL-6, IL-10 and MCP-1 in the plasma. These cytokines might play a role in increases the permeability of BBB to allow the virus into the CNS.

For viruses, efficient elimination of the infection requires a proinflammatory host response and development of Type I immunity [29,30]. Cytokines act both destructive and protective agents in virus infection. The reduction in CD4+, CD8+ and CD19+ cells at 72 h PI might be due to the high secretion of these proinflammatory cytokines. Role of these cytokines involved in apoptosis of lymphocytes were well described by several authors. In seasonal influenza and H5N1 infection, macrophages and alveolar cells produce high level of inflammatory cytokines leading to the apoptosis of lymphocytes [31,32]. Laboratory study on mice also confirmed this result [33]. Bennet *et al*, 2001 in their research concluded that TNFα enhanced the *Fas *mediated apoptosis of unactivated T cells through decrease intracellular levels of FLIP and increased production of proapoptotic molecules like Bax. This effect was mediated through TNFR2 receptor [34]. TNF signalling through TNFR2 sensitize lymphoid cells for *Fas *mediated apoptosis. TNFα mediated reduction of lymphocytes also reported in disease like Feline infectious peritonitis, African swine fever, HIV etc [35-37]. Secretion of TNFα is also reported in clinical human case of Chandipura virus [40].

This reduction of lymphocyte along with other factors like secretion of proinflammatory cytokines during infection might leads to suppression of T cell proliferation. In acute toxoplama infection the secretion of IFNγ and IL-10 involved significantly the Con A and toxoplasma antigen mediated suppression of lymphocytes [38]. In Lymphocytic choriomeningitis virus (LCMV) in mice it was concluded that T-lymphocyte unresponsiveness should be related to an inherent proliferative defect subsequent to T-cell activation and IL-2 receptor expression [39]. Mechanism behind the suppression of T cell proliferation is still inconclusive.

## Conclusion

From this study we concluded that the secretion of high quantity of proinflammatory cytokines in mice during infection might leads to pathogenesis. Early production of IgM could clears the virus from circulation but its inability of clear the virus from CNS paves the way for uninterrupted replication in brain. This leads to neuropathology and mortality. Once the virus entered the CNS even passive immunization may not protect the mice. Inhibition of specific cytokines like TNFα or overall inhibition of these cytokines might help to reduce the pathogenesis.

## Methods

### Virus

Chandipura virus isolate 03-4267, originally isolated in 2003 in Andra Pradesh, India. The virus was propagated in Vero E6 cells. The titer of virus was found to be 10^7 ^TCID_50 _per ml.

### Animal experimentation

Young Swiss albino mice always kept along with mother upto weaning (16 day) for milk feeding. Each group consists of eight young once along with mother. The group of mice in the age of 13–14 day old was used in all the experiments. All the experiments carried out with proper permission obtained from Institute Animal Ethical Committee (IAEC). The mice were experimentally infected with 50 μl of Chandipura virus through intra venous (I/V) route. The control mice were injected with PBS. Blood and brain was collected at 0, 24, 48 and 72 h PI. Mice were perfused transcardially with 20 ml of PBS (pH 7.4) and the brain was collected and frozen immediately at -80°C until use. Blood was collected intra orbitaly before sacrifice and the sera were separated and frozen for further use. Plasma was separated by centrifugation from the blood collected in EDTA. The frozen tissues were freeze thawed and 10% suspensions in PBS were prepared by homogenization. The homogenized suspension was clarified at 3000 rpm for 10 min and the supernatant was used for titration.

### Titration of virus in blood and brain

Tenfold dilution of sera and brain suspensions were prepared in serum free DMEM medium. Confluent monolayer of Vero E6 cells in 96 well flat bottom microtiter plate was infected in triplicate for each dilution. The plate was incubated at 37°C with 5% CO_2 _tension. At 48 h PI, the plate was stained with 0.1% crystal violet in formal saline and the virus titer was expressed as TCID_50_/ml [22].

### Assay of blood brain barrier integrity

BBB integrity was checked with Evan's blue dye exclusion test [23]. Briefly at different time point's three mice from infected as well as control groups were injected intravenously with 100 μl of 2% Evan's blue (Sigma) in PBS. One hour later, mice were sacrificed and transcardially perfused with 20 ml of normal saline. Brains were removed and photographed.

### IgM and IgG kinetics

IgM and IgG capture ELISA was done following procedure similar to the IgM capture ELISA used for human [24]. With only modification that coating was done with anti mouse IgM and Goat anti mouse IgG (γ chain specific) (Calbiochem) was used as a capture antibody for IgG capture ELISA. The cut off value was set by average plus 3 standard deviation of O.D from uninfected negative control mice. The O.D above cut off value was considered to be positive.

### Passive immunization

Mice were divided into five groups. Group I mice received 50 μl of rabbit anti Chandipura antibody (Neutralizing titer of 10240) through I/P route at 24 h before infection. Group II mice received antibody on that day of infection and group III mice received at 24 h PI. Group IV mice were kept as a virus control and group V was kept as a PBS control. Antibody treatment was followed upto 72 h PI invariably in all group of mice except virus control. All mice were infected with virus through I/V route. The mortality pattern was observed for 8 days PI.

### Staining for analysis of cell phenotype

Phenotypes of cells were determined by using monoclonal antibodies (Mab) against CD4, CD8 and CD19 receptors (BD Pharmingen, eBioscience). Antibodies were conjugated to various fluorochrome like fluorecein isothiocyanate (FITC), phycoerythrin (PE), or PE cyanine5.5 conjugate, and corresponding immunoglobulin G (IgG) matched isotype control antibodies were used to set baseline values for analysis markers. For surface staining, appropriate concentration of combinations of multible Mabs were mixed with cells and treated as described by the manufacturer (BD Pharmingen).

### Flowcytometry analysis

Acquisition and analysis were done using FACScalibur (BD Bioscience). For analysis of lymphocytes forward versus side scatter was used for gating. Acquisition and analysis were performed using cell quest software (BD Bioscience).

### Lymphocyte Transformation Test

Splenocyte single cell suspension was prepared by pipetting the spleen in 5 ml of RPMI 1640 supplemented with 10% FCS, 25 mM HEPES and 5 × 10^-5 ^M β-mercato ethanol and layered on to the histopaque (1.083 gm/ml) (Sigma). The mononuclear cell population was removed after centrifugation and washed twice in RPMI 1640. The cell concentration was adjusted to 1 × 10^6^/ml in RPMI 1640 medium. One hundred of diluted cell suspension was dispensed into 96-well flat bottom culture plate. Mitogens Concanavalin A (Con A) and lipopolysaccaride (LPS)(Sigma) derived from *E. coli *and BPL inactivated Chandipura virus antigen were added 0.1 μg, 0.01 μg and 0.2 μg final concentration respectively in specified wells. After incubation of 72 h at 37°C with 5% CO_2_, the proliferation was determined by MTT dye reduction method [25]. Briefly, 10% of (3-(4, 5 diamethyl-2-thiazolyl) 2,5-diphenyl-2H-tetrazolium) (MTT) (5 mg/ml) was added to each well and plates were incubated at 37°C in CO_2 _humid atmosphere for 4 h. The blue formazan precipitate was dissolved in 20% SDS in 50% DMF and its optical density was measured in 540 nm and a reference wavelength of 650 nm using ELISA Reader (Biorad). Each mitogen and the antigen were tested in quadruplicate wells. The stimulation index (SI) was calculated by the following equation:

Proliferation = [(Stimulated OD - Unstimulated OD)/Unstimulated OD]

The stimulation index of two differences between control and infected mice was considered as a cut off value. Those mice showing cut off value above than control mice was considered proliferation and the value below was considered as suppression.

### Cytometric Bead assay (Mouse pro inflammatory cytokines)

The level of IL-12p70, TNFα, IFNγ, MCP-1, IL-10 and IL-6 cytokines in the plasma was quantitated by Cytometric Bead assay for Mouse inflammatory cytokines according to the manufacurer's instructions (BD pharmingen). The minimum to maximum sensitivity of this assay is 0–5000 pg/ml. All the six cytokines were simultaneously quantitated from 50 μl of undiluted plasma. The quantity of different cytokines in plasma was compared with uninfected age matched control and expressed as pg/ml.

### Statistical analysis

Antibody kinetics was analysed by Mann-Whitney rank test. Level of different immune cells in blood, cytokines and proliferation of lymphocytes between control and infected mice were analysed by student's *t*-test. The *p *values were mentioned in respective places.

## Competing interests

The authors declare that they have no competing interests.

## Authors' contributions

AB designed the study, performed the experiments and drafted the manuscript. ACM participated in design and helped to draft the manuscript. All the authors read and approved the final manuscript.

## References

[B1] Chadha MS, Arankalle VA, Jadi RS, Joshi MV, Thakare JP, Mahadev PVM, Mishra AC (2005). An outbreak of Chandipura virus encephalitis in the eastern districts of Gujarat state, India. Am J Trop Med Hyg.

[B2] Sokhei CH, Obukhova VR (1984). Susceptibility of laboratory animals to the Chandipura and isfahan viruses. Vopr Virusol.

[B3] Bhatt PN, Rodrigues FM (1967). Chandipura virus: a new arbovirus isolated in India from patients with febrile illness. Indian J Med Res.

[B4] Jortner BS, Bhatt PN, Solitare GB (1973). Experimental Chandipura virus infection in mice. I. Virus assay and light microscopic studies with emphasis on neuropathologic observations. Acta Neuropathologica.

[B5] Fenner FJ (1968). The pathogenesis of viral infections: the influence of age on resistance to viral infections. The biology of animal viruses.

[B6] Sigel MM (1952). Influence of age on susceptibility to virus infection with particular reference to laboratory animals. Annu Rev Microbiol.

[B7] Griffin DE, Levine B, Tyor WR, Tucker PC, Hardwick JM (1994). Age-dependent susceptibility to fatal encephalitis: alphavirus infection of neurons. Arch Virol Suppl.

[B8] Levine B (2002). Apoptosis in viral infections of neurons: a protective or pathologic host response?. Curr Top Microbiol Immunol.

[B9] Oliver K, Scallan M, Dyson H, Fazkerly J (1997). Susceptibility to a neurotropic virus and its changing distribution in the developing brain is a function of CNS maturity. J Neurovirol.

[B10] Oliver K, Fazakerley J (1998). Transneuronal spread of Semliki forest virus in the developing mouse olfactory system is determined by neuronal maturity. Neuroscience.

[B11] Ogata A, Nagashima K, Hall WW, Ichikawa M, Kimura-Kuroda J, Yasui K (1991). Japanese Encephalitis Virus Neurotropism Is Dependent on the Degree of Neuronal Maturity. J Virol.

[B12] Tardieu M, Powers ML, Weiner HL (1983). Age dependent susceptibility to reovirus type 3 encephalitis: role of viral and host factors. Ann Neurol.

[B13] Fazakerley JK, Allsopp TE (2001). Programmed cell death in virus infections of the nervous system. Curr Top Microbiol Immunol.

[B14] Hackbarth SA, Reinarz AB, Sagik BP (1973). Age-dependent resistance of mice to sindbis virus infection: reticuloendothelial role. J Reticuloendothel Soc.

[B15] Sabin AB (1941). Constitutional barriers to involvement of the nervous system by certain viruses, with special reference to the role of nutrition. J Pediatr.

[B16] Kunin CM (1962). Virus-tissue union and the pathogenesis of enterovirus infections. J Immunol.

[B17] Heinenberg H, Gold E, Robbins FC (1964). Differences in interferon content in tissues of mice of various ages infected with Coxsackie B1 virus. Proc Soc Exp Biol Med.

[B18] Weiner LP, Cole GA, Nathanson N (1970). Experimental encephalitis following peripheral inoculation of West Nile virus in mice of different ages. J Hyg (Lond).

[B19] Overman JR, Kilham L (1953). The inter-relation of age, immune response and susceptibility to mumps virus in hamsters. J Immunol.

[B20] Trgovcich J, Aronson JF, Eldridge JC, Johnston RE (1999). TNFα, interferon and stress response induction  as a function of age-related susceptibility to fatal Sindbis virus infection of  mice. Virology.

[B21] Labrada L, Liang XH, Zheng W, Johnston C, Levine B (2002). Age dependent resistance to lethal alphavirus encephalitis in mice: analysis of gene expression in the central nervous system and identification of a noval interferon inducible protective gene, Mouse ISG12. J Virol.

[B22] Reed LJ, Muench H (1938). A simple method of estimating fifty percent end points. Am J Hyg.

[B23] Mikawa S, Kinouchi H, Kamii H, Gobbell GT, Chen SF, Carlson E, Epstein CJ, Chan PH (1996). Attenuation of acute and chronic damage following traumatic brain injury in copper, zinc-super oxide dismutase transgenic mice. J Neurosurg.

[B24] Rao BL, Basu A, Wairagkar NS, Gore MM, Arankalle VA, Thakare JP, Jadi RS, Rao KA, Mishra AC (2003). A large outbreak of acute encephalitis with high fatality rate in children in Andrapradesh, India, in associated with Chandipura virus. Lancet.

[B25] Mosmann T (1983). Rapid colorimetric assay for cellular growth and survival: application of proliferation and cytotoxicity assays. J Immunol Meth.

[B26] Amor S, Scallan MF, Morris MM, Dyson H, Fazakerley JK (1996). Role of immune responses in protection and pathogenesis during Semliki Forest virus encephalitis. J Gen Virol.

[B27] Tsao N, Hsu HP, Wu CM, Liu CC, Lei HY (2001). Tumor necrosis factor-α causes an increase in blood-brain barrier permeability during sepsis. J Med Microbiol.

[B28] Dickstein JB, Moldofsky H, Hay JB (2000). Brain-blood permeability: TNF-α promotes escape of protein tracer from CSF to blood. Am J Physiol Regul Integr Comp Physiol.

[B29] Lucey DR, Clerici M, Shearer GM (1996). Type 1 and type 2 cytokine dysregulation in human infectious, neoplastic, and inflammatory diseases. Clin Microbiol Rev.

[B30] Romagnani S (1997). The Th1/Th2 paradigm. Immunol Today.

[B31] Cheung CY, Poon LL, Lau AS, Luk W, Lau YL, Shortridge KF, Gordon S, Guan Y, Peiris JS (2002). Induction of proinflammatory cytokines in human macrophages by Influenza A (H5N1) viruses: a mechanism for the unusual severity of human disease?. Lancet.

[B32] Chen MC, Cheung CY, Chui WH, Taso SW, Nicholls JM, Chan RW, Long HT, Poon LL, Guan Y, Peiris JS (2005). Proinflammatory cytokine response induced by influenza A (H5N1) viruses in primary human alveolar and bronchial epithelial cells. Resp Res.

[B33] Tumpey TM, Lu X, Morken T, Zaki SR, Katz JM (2000). Depletion of lymphocytes and diminished cytokine production in mice infected with a highly virulent influenza A (H5N1) virus isolated from humans. J Virol.

[B34] Bennett DE, Griffith ST, Herndon JM, Barreiro R, Tschopp J, Ferguson TA (2001). Regulation of Fas Ligand-Induced apoptosis by TNF. J Immunol.

[B35] Takano T, Hohdatsu T, Hashida Y, Kaneko Y, Tanabe M, Koyama H (2007). A possible involvement of TNF-alpha in apoptosis induction in peripheral blood lymphocytes of cats with feline infectious peritonitis. Vet Microbiol.

[B36] Sanchez-Cordon PJ, Nunez A, Salguero FJ, Pedrera M, Fernandez De Marco M, Gomez-Villamandos JC (2005). Lymphocyte apoptosis and thrombocytopenia in spleen during classical swine fever: role of macrophages and cytokines. Vet Pathol.

[B37] Alimonti JB, Blake Ball T, Fowke KR (2003). Mechanisms of CD4+ T lymphocyte cell death in human immunodeficiency virus infection and AIDS. J Gen Virol.

[B38] Candolfi E, Hunter CA, Remington JS (1995). Roles of gamma interferon and other cytokines in suppression of spleen cell proliferative response to Concanavalin A and Toxoplasma antigen during acute toxoplasmosis. Infect Immun.

[B39] Saron MF, Shidani B, Nahori MA, Guillon JC, Truffa-Bachi P (1990). Lymphocytic chroriomeningitis virus-induced immnodeprssion: inherent defect of B and T lymphocytes. J Virol.

[B40] Tripathy A, Balaji S, Rao N, Thakare JP, Mishra AC, Arankalle VA (2005). Cytokine levels in Chandipura virus associated encephalopathy in children. Scand J Infect Dis.

